# Nutritional quality of dysphagia-oriented products sold on the Italian market

**DOI:** 10.3389/fnut.2024.1425878

**Published:** 2024-07-03

**Authors:** Giorgio Innocenzo Ascrizzi, Daniela Martini, Laura Piazza

**Affiliations:** ^1^Department of Environmental Science and Policy (ESP), Università degli Studi di Milano, Milan, Italy; ^2^Department of Food, Environmental and Nutritional Sciences (DeFENS), Università degli Studi di Milano, Milan, Italy

**Keywords:** dysphagia, dysphagia-oriented products, texture-modified foods, nutritional quality, food labeling, foods for special medical purposes

## Abstract

**Introduction:**

Dysphagia is a condition characterized by swallowing difficulties that affects an estimated 8% of the population. Management of dysphagia often requires the use of specially formulated food products that are easier to swallow, while still meeting the nutritional needs of the patient. Despite the growing market for dysphagia-oriented products, there is a compelling need for comprehensive evaluations of their nutritional quality to ensure that they adequately support the health and well-being of this vulnerable population. The aims of this study were: (i) to investigate the nutritional composition of different dysphagia products currently sold in Italy, from several leading healthcare companies, by collecting the nutritional information on their packaging; (ii) to compare their energy, nutrient and salt content per 100 g and serving.

**Methods:**

A total of 70 items, available in the Italian online market were included in the analysis.

**Results:**

The data showed a wide difference among the six categories of dysphagia-oriented products. Salt content was found to be very high, with medium (>0.3 g/100 g but <1 g/100 g) and high (≥1 g/100 g) content found in 17 and 51% of products, respectively. Overall, the results show high variability in nutritional composition among dysphagia-oriented products currently on the market.

**Discussion:**

The high presence of salt in more than half of the products raises a critical issue, as it is not in accordance with WHO guidelines and especially with the clinical situation of the dysphagia patient. This research seeks to provide valuable insights into the adequacy of these products in meeting the dietary requirements of individuals with dysphagia, thereby guiding toward more informed and suitable food choices.

## 1 Introduction

Dysphagia is a significant health problem affecting an estimated 8% of the global population, with a pronounced prevalence among the elderly (1). Dysphagia is defined as the difficulty or inability to swallow, which can result from various diseases (e.g., neuromuscular disorders, brain injury, stroke) or related to natural aging process. Moreover, dysphagia can cause choking or aspiration when swallowing thin liquids or solid foods. The implications of dysphagia are significant for patients’ lives, as the risk of choking or aspiration can generate considerable anxiety and potentially lead to suboptimal nutritional and hydration status, due to voluntary reduction in intake. Especially, older subjects with dysphagia have increased malnutrition compared with those without dysphagia (2). Patients with dysphagia have fewer options in their choice of food types and tend to prefer soft-solid foods that are easier to swallow, regardless of nutrient content and caloric intake. All these factors have been associated with lower quality of life for dysphagic patients (3).

The modification of texture and viscoelastic properties of foods and liquids is an effective strategy to promote a safe and easily swallowing. By thickening fluids, oropharyngeal transit time is increased, and a more cohesive bolus is created, allowing greater muscle adaptation, and thus compensating for the swallowing deficit (4). The degree of texture modification and liquid thickening are internationally standardized by the International Dysphagia Diet Standardization Initiative (IDDSI), which provides categorization of food textures applicable to neonates, infants, children, and adults with dysphagia (1). The IDDSI framework includes 5 levels of beverage thickness (levels 0–4) and 5 levels of food texture (levels 3–7). The beverage part includes the thin level (level 0) and 4 levels of increasing thickness, while the food part includes the normal/easily chewable level (level 7) and 4 levels of textural modification.

However, sensory acceptance of dysphagia-oriented products is often compromised. This is related to the altered texture which can detract from the visual appeal and palatability of the food, sometimes altering its smell, shape, and taste. Consequently, patients may eat less because of dissatisfaction with sensory qualities or fear of eating, as these products are often poorly acceptable due to factors such as loss of flavor and inadequate texture, which characterize these preparations (5). The development of products tailored for individuals with dysphagia is crucial for enhancing their quality of life. It is essential to balance textural and sensory modifications with nutritional integrity to ensure these foods meet the dietary needs of this population. Current dysphagia diets are often lacking in fruits, vegetables, and whole grains, while being high in fat, sugar, and salt. To make dysphagia-oriented foods more appealing, manufacturers frequently add salt, sugar, and artificial flavors. This can result in increased intake of sodium and sugars, which is especially concerning for older patients who may also be managing conditions such as hypertension and diabetes (6). This is not suitable for long-term consumption and leads to a demand for nutritional improvement of foods intended for patients with dysphagia.

In the European Union (EU), dysphagia-oriented products are regulated by Regulation No. 609/2013 (7), for the classification of Foods for Special Medical Purposes (FSMP). Dysphagia-oriented products are categorized as FSMP, which is a group of products intended for the dietary management of specific groups of patients with deficits that need to be medically supervised (8). In addition, product nutritional information is available to the consumer on the food label, also in accordance with Regulation No. 609/2013 (7). Moreover, as stated by Regulation No. 128/2016, the formulation of FSMP must be safe, beneficial, and effective in meeting the specific nutritional needs of the people for whom they are intended, as demonstrated by generally accepted scientific data (9). However, Regulation No. 128/2016 does not report any directive regarding nutritional composition and salt content of FSMP.

Patients should be guided toward more informed and conscious food choices that can lead to better eating behaviors and to better meeting the nutritional requirements. Improving health and nutrition literacy is essential for ensuring that dysphagic patients make informed dietary choices. In this context, food labels play a crucial role in enhancing nutrition literacy by providing essential information that helps consumers make healthier choices. For dysphagia-oriented products, indicating the texture level according to the IDDSI level can help ensure the product is suitable for the patient’s swallowing ability (10).

It is of our interest to investigate the nutritional quality of dysphagia-oriented products sold in the Italian market by collecting the nutritional information reported on their packaging. Based on these premises, the aim of the present study is to systematically investigate the nutritional composition of dysphagia-oriented products sold in the Italian market, also to identify characteristics that could improve the nutritional and sensory composition of these products. Following this initial evaluation based on nutritional characteristics, future studies will focus on the texture and viscoelastic properties of commercially available dysphagia-oriented products, according to the IDDSI framework. This will provide a more appropriate categorization and a comprehensive understanding of how these products can be improved to better meet the needs of individuals with dysphagia, ultimately leading to the development of more appealing food options.

## 2 Materials and methods

### 2.1 Food product selection

In the present cross-sectional study, information on dysphagia-oriented products was obtained from manufacturers’ websites or, where absent, from major public-useable pharmaceutical retailers in the Italian online market. The online search was conducted from October 2023 to December 2023. All dysphagia-oriented products bearing mandatory food information on the package, as required by Regulation No. 1169/2011 (11), were included. Conversely, products with the following characteristics were excluded: (i) not available online during the data collection phase; (ii) with partial package images; and (iii) with an incomplete list of ingredients.

### 2.2 Data collection and analysis

For all selected products, full package images data were collected. Qualitative and quantitative data indicated on the label of all products were recorded, including: brand name, descriptive name, ingredients, energy (kcal/100 g), total lipids (g/100 g), saturated fatty acids (g/100 g), total carbohydrates (g/100 g), sugars (g/100 g), fiber content (g/100 g), proteins (g/100 g), and salt (g/100 g), as done in previous studies (12–14). Once these values were retrieved, data on energy and nutrient content per standard portion were also presented, using as portion size the amount in grams reported on each label.

The descriptive name, along with the main ingredient contained in the meal, were used to classify products into six categories: (i) carbohydrate-rich foods, (ii) protein-rich foods, (iii) fruits and vegetables, (iv) desserts, (v) breakfast meals, and (vi) thickened water. Where possible, categorization was done according to food groups while desserts and breakfast meals were categorized otherwise. In particular, the distinction between desserts and breakfast products was made based on the product characteristics, i.e., the desserts category contains cake and spoon cake substitute products, while the breakfast category includes milk and cereal and/or oat products. After data collection, a dataset was created (Microsoft Windows Excel 2017 software) grouping products into the aforementioned six categories of interest based on descriptive name and the food group.

For the analysis of salt content, products were classified as following: “very low salt content” (<0.10 g of salt/100 g) and “low salt content” (<0.3 g of salt/100 g), following the Regulation No. 1924/2006 (15). The remaining products were instead classified as “medium salt content” (>0.3 but <1 g of salt/100 g) and “high salt content” (≥1 g of salt/100 g) as previously reported (14).

### 2.3 Statistical analysis

The Statistical Package for Social Sciences software (IBM SPSS Statistics, Version 29.0, IBM corp., Chicago, IL) and OriginPro software (version 9.0, Stat-Ease Company, Northampton, MA, United States) were used to perform the statistical analysis, with a significance level set at *p* < 0.05. The normality of data distribution was first verified through the Kolmogorov–Smirnov test, then rejected. The variables were then expressed as median and interquartile range. The Kruskal-Wallis test was employed to determine if there were significant differences between the categories for each macronutrient. The test revealed significant differences for all macronutrients (*p* < 0.05). Therefore, Dunn’s test, adjusted with Bonferroni correction for multiple comparisons, was conducted to identify specific categories with significant differences in macronutrient content.

## 3 Results

### 3.1 Nutritional content per 100 g

A total of 70 different meal substitutes were analyzed. Protein-rich foods represent the most abundant category (*n* = 19), followed by fruits and vegetables (*n* = 13), desserts (*n* = 11), carbohydrate-rich foods (*n* = 10), breakfast meals (*n* = 9), and thickened water (*n* = 8). All categorized products belonged to one of the following product types: ready to eat (*n* = 21) or freeze-dried (*n* = 49). Further differentiation consisted in the portioning of the products surveyed. The majority of products were presented as already portioned as single-serving (*n* = 52), while the remainder were not portioned, but sold in 1.0 kg packages (*n* = 18).

The nutritional characteristics of all the 70 retrieved items, also divided into the six categories, are reported in Table 1. Overall, carbohydrates were the most abundant macronutrients (45.5%), followed by protein content (18.7%) and lipids (14.0%).

**Table 1:** Energy and nutritional composition across the dysphagia-oriented products.

	Dysphagia-oriented products						
	All (*n* = 70)	Carb-rich foods (*n* = 10)	Protein-rich foods (*n* = 19)	Fruits and Vegetables (*n* = 13)	Desserts (*n* = 11)	Breakfast meals (*n* = 9)	Thickened water (*n* = 8)
Energy (Kcal/100 g)	424	444 ^a,b^	431 ^a^	441 ^a,b^	171 ^b^	392 ^a,b^	5.0 ^c^
	171–450	419–458	424–445	245–452	142–471	375–420	3.7–6.0
Lipids (g/100 g)	14.0	16.4 ^a^	15.0 ^a,b^	15.3 ^a,b^	7.4 ^a,b^	8 ^b^	0 ^c^
	4.8–16.2	15.3–16.9	13.6–19.3	9.3–15.7	4.7–18.9	1.2–10.8	0.0–0.5
SFA (g/100 g)	2.3	4.6 ^a^	3.6 ^a^	3.5 ^a^	1.3 ^a^	1.5 ^a^	_
	0.9–7.1	3.1–10.1	2.1–8.0	1.0–7.7	1.0–7.4	0.3–3.2	
Carb (g/100 g)	45.5	43.5 ^b^	45.0 ^b^	49.0 ^b^	41.0 ^b^	67.0 ^a^	0.8 ^c^
	20–56.8	35.3–52.6	36.5–51.3	29.1–57.4	14.0–60.0	59.0–83.0	0.5–1.2
Sugar (g/100 g)	5.5	5.5 ^b^	4.7 ^b^	5.5 ^b^	9.0 ^b^	13.0 ^a^	_
	3.0–9.4	3.9–6.5	3.1–6.2	5.2–6.2	5.4–11.9	9.2–22.0	
Fiber (g/100 g)	3.1	5.0 ^a^	4.5 ^a^	4.4 ^a^	3.1 ^a^	2.6 ^a^	1.0 ^b^
	1.3–6.5	1.4–11.0	1.3–7.5	2.6–6.0	1.5–3.2	1.7–5.4	0.7–1.2
Proteins (g/100 g)	18.7	22.9 ^a^	22.0 ^b^	19.4 ^a^	9.5 ^a^	15.0 ^a^	0 ^c^
	8.8–22.6	18.0–23.8	20.3–24.0	9.6–20.7	8.8–18.3	8.2–19.1	0.0–0.5
Salt (g/100 g)	1.0	2.5 ^a^	2.0 ^a,b^	1.10 ^a,b^	0.4 ^b^	0.3 ^b,c^	0.06 ^c^
	0.2–2.5	1.7–3.4	1.1–3.0	0.2–2.6	0.2–0.7	0.03–0.45	0.05–0.07

Values are expressed as median (25th–75th percentile). For each row, different uppercase letters indicate a significant difference for each parameter (*p* < 0.05; Dunn test). SFA, saturated fatty acid; Carb, carbohydrate.

Among the different categories, carbohydrate-rich foods had the highest total median energy (total median value of 444 (419–458) kcal/100 g), which was not significantly different when compared to fruits and vegetables (441 (245–452) kcal/100 g), protein-rich foods (431 (424–445) kcal/100 g), and breakfast meals (392 (375–420) kcal/100 g). Significative differences were found between these categories and desserts (171 (142–471) kcal/100 g) and thickened water (5.0 (3.7–6.0) kcal/100 g).

Similarly, carbohydrate-rich foods showed the highest total lipid content (total median value of 16.4 (15.3–16.9) g/100 g) with no significant differences when compared to fruits and vegetables (15.3 (9.3–15.7) g/100 g), protein-rich foods (15.0 (13.6–19.3) g/100 g), and desserts (7.4 (4.7–18.9) g/100 g). Significative differences were found between carbohydrate-rich foods and breakfast meals (8.0 (1.2–10.8) g/100 g) and thickened water (0.0 (0.0–0.5) g/100 g).

Besides, saturated fatty acids (SFA) content was highest in carbohydrate-rich foods (total median value of 4.6 (3.1–10.1) g/100 g), followed by protein-rich foods (3.6 (2.1–8.0) g/100 g), fruits and vegetables (3.5 (1.0–7.7) g/100 g), breakfast meals (1.5 (0.3–3.2) g/100 g) and desserts (1.3 (1.0–7.4) g/100 g); where no significative differences were found between these groups.

The carbohydrate content was significantly higher in breakfast meals (total median value of 67.0 (59.0–83.0) g/100 g) compared to all the other categories. However, with regard to the other categories, no significant difference was found between fruits and vegetables (49.0 (29.1–57.4) g/100 g), protein-rich foods (45.0 (36.5–51.3) g/100 g), carbohydrate-rich foods (43.5 (35.3–52.6) g/100 g), and desserts (41.0 (14.0–60.0) g/100 g), except for thickened water (0.8 (0.5–1.2) g/100 g).

Similarly, breakfast meals category showed the highest sugar content (13.0 (9.2–22.0) g/100 g), which was statistically significant when compared to the other categories. As for the other categories, instead, no significant difference was found between desserts (9.0 (5.4–11.9) g/100 g), carbohydrate-rich foods (5.5 (3.9–6.5 g) g/100 g), fruits and vegetables (5.5 (5.2–6.2) g/100 g), and protein-rich foods (4.7 (3.1–6.2) g/100 g).

Carbohydrate-rich foods were found to have the highest fiber content (total median value of 5.0 (1.4–11.0) g/100 g), followed by protein-rich foods (4.5 (1.3–7.5) g/100 g) and fruits and vegetables (4.4 (2.6–6.0) g/100 g), but with no significant differences when compared to desserts (3.1 (2.0–3.2) g/100 g) and breakfast meals (2.6 (2.0–5.0) g/100 g), except for thickened water (1.0 (0.7–1.2) g/100 g).

The highest protein content was found in carbohydrate-rich foods (total median value of 22.9 (18.0–23.8) g/100 g), which was not statistically significantly different to fruits and vegetables (19.4 (9.6–20.7) g/100 g), breakfast meals (15.0 (8.4–19.0) g/100 g) and desserts (9.5 (9.2–17.2)); except for protein-rich foods (22.0 (20.3–24.0) g/100 g) and thickened water (0.0 (0.0–0.5) g/100 g).

### 3.2 Focus on salt content

As reported in Table 1, the dysphagia-oriented products with the highest salt content per 100 g were carbohydrate-rich foods (2.5 (1.7–3.4) g/100 g), protein-rich foods (2.0 (1.1–3.0) g/100 g) and fruits and vegetables (1.1 (0.2–2.6) g/100 g).

**Figure 1:**
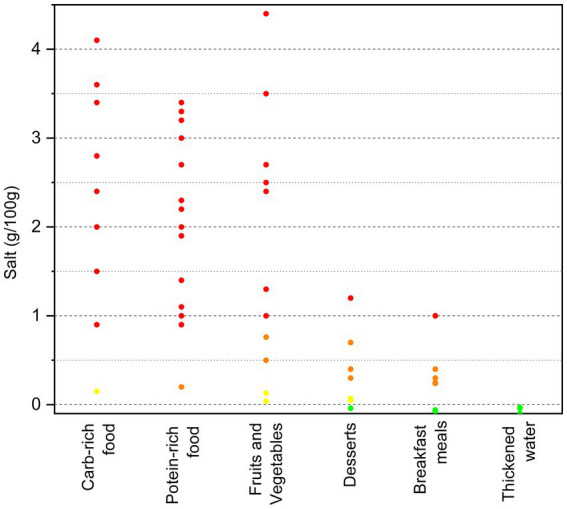
Salt content (g/100 g) of the categorized dysphagia-oriented products. Products were classified as: green = very low salt content (<0.10 g of salt/100 g) and yellow = low salt content (<0.3 g of salt/100 g), following the Regulation No. 1924/2006 (15). The remaining products were instead classified as: orange = medium salt content (>0.3 but <1 g of salt/100 g) and red = high salt content (≥1 g of salt/100 g), as previously reported (14).

The salt content of all the categorized dysphagia-oriented products is shown in Figure 1. Very low content of salt (<0.10 g/100 g) was found in the 19% of the products, while low content of salt (<0.3 g/100 g) was reported by 13% of the products. A medium (>0.3 g/100 g but <1 g/100 g) and high (≥1 g/100 g) category of salt content was reported for 17 and 51%, respectively.

### 3.3 Nutritional content per serving

**Figure 2:**
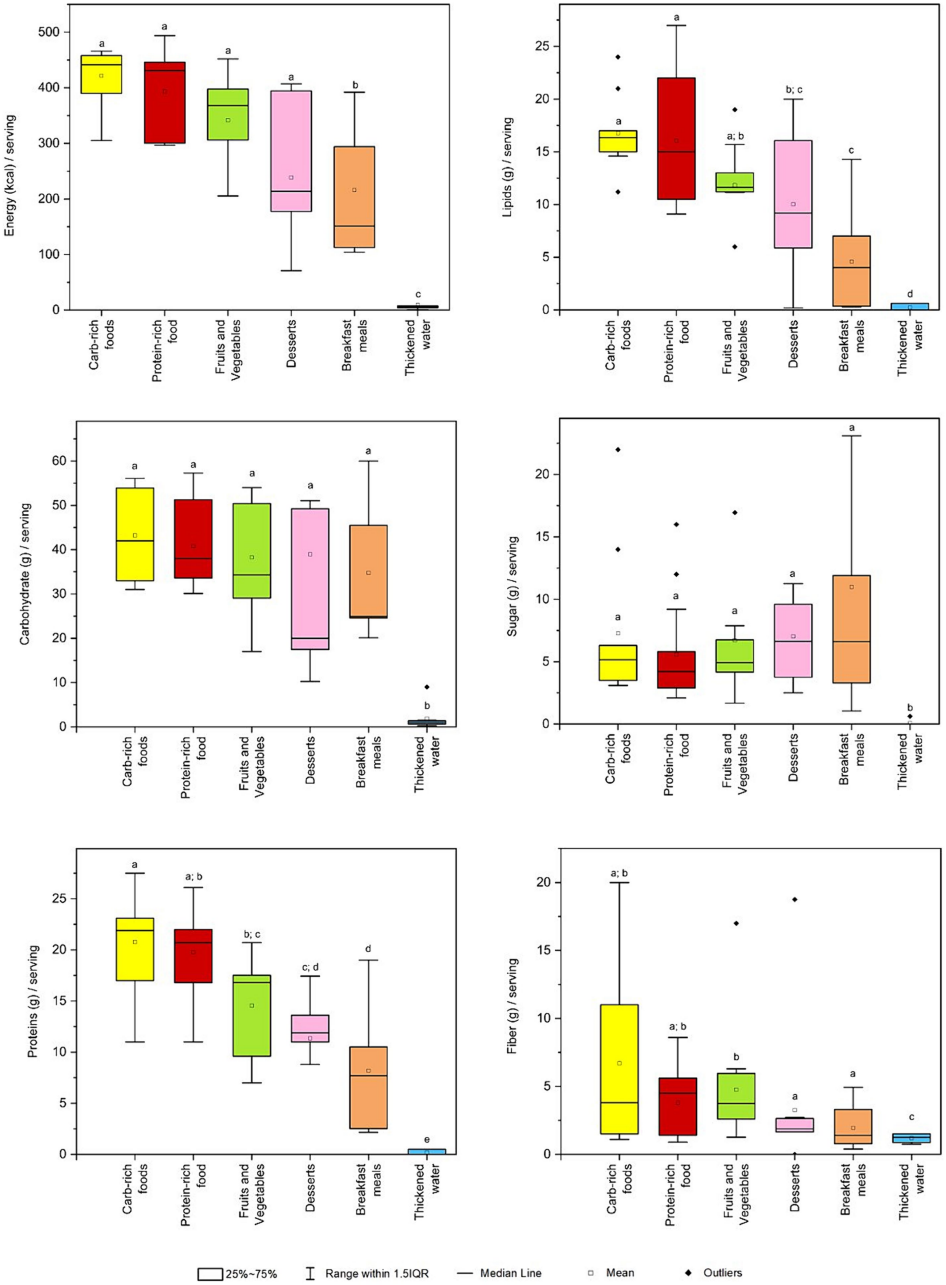
Box plot for energy and nutrition facts per standard serving across categories of dysphagia-oriented products. Standard serving was retrieved for every product as stated on the label. For each category, different upper letters indicate a significant difference among the different dysphagia-oriented products categories (*p* < 0.05; Dunn test). Carb, carbohydrate.

To further analyze the nutritional content of all types of dysphagia-oriented products, an additional evaluation of the nutritional data (energy, nutrients, and salt) per serving was performed. The standard serving size for each product was determined according to the label. However, each category has its own standard serving size, and in some cases, a different serving size was found between ready-to-eat and freeze-dried product to be prepared. The servings taken into analysis are shown in Table 2. Moreover, it is important to note that 74% of the products were sold already portioned as single-serving, while the remaining 26% were not single-served but sold in 1.0 Kg packs. The results related to the nutritional characteristics for serving are shown in Figure 2.

**Table 2:** Servings per each category, according to the labeling.

	Serving size	
Category	Ready to eat	Dry
Carbohydrate-rich foods	300 g (*n* = 1)	100 g (*n* = 9)
Protein-rich foods	300 g (*n* = 2)	70 g (*n* = 6) 100 g (*n* = 11)
Fruits and vegetables	125 g (*n* = 2) 150 g (*n* = 2)	70 g (*n* = 2) 85 g (*n* = 5) 100 g (*n* = 2)
Desserts	125 g (*n* = 6)	85 g (*n* = 5)
Breakfast meals	–	70 g (*n* = 7) 85 g (*n* = 1) 100 g (*n* = 1)
Thickened water	125 g (*n* = 8)	–

Servings divided by category. For each category, if applicable, data is provided for both ready-to-eat products and products to be prepared. Within the same category, some products have different portions, which reflect the different manufacturer brands.

Regarding the energy content per reported serving on the label, it was significantly higher for carbohydrate-rich foods (total median value of 442 (396–458) kcal/serving), protein-rich foods (431 (301–445) kcal/serving), fruits and vegetables (368 (306–398) kcal/serving) and desserts (214 (179–329) kcal/serving) compared to the other categories. Significative differences were found between these categories and breakfast meals (151 (113–294) kcal/serving) and thickened water (6.3 (4.6–7.5) kcal/serving).

Total lipids were found to be significantly higher for carbohydrate-rich foods (total median value of 16.4 (15.2–16.9) g/serving), protein-rich foods (15.0 (10.9–19.6) g/serving) and fruits and vegetables (11.6 (11.2–13.0) g/serving). In addition, no significant differences were found between the fruits and vegetables and desserts (9.2 (5.9–15.6) g/serving) categories, and between the desserts and the breakfast meals (4.0 (0.4–7.0) g/serving) categories.

Carbohydrates were predominant among carbohydrate-rich foods (total median value of 42.0 (34.5–52.6) g/serving), protein-rich foods (38.0 (33.8–48.0) g/serving), and fruits and vegetables (36.4 (32.2–50.4) g/serving), followed by breakfast meals (24.9 (24.6–45.5) g/serving) and desserts (20.0 (17.5–48.5) g/serving). No significative difference were found between these categories, except for thickened water (0.9 (0.6–1.3) g/serving).

Sugar contents were higher among desserts (total median value of 6.6 (3.8–9.5) g/serving), breakfast meals (6.6 (3.3–11.9) g/serving) and carbohydrate-rich foods (5.2 (3.8–6.2) g/serving), followed by fruits and vegetables (4.4 (3.9–4.9) g/serving) and protein-rich foods (4.2 (3.1–5.5) g/serving). No significant difference was found between categories except for thickened water.

Protein content was higher in carbohydrate-rich foods (total median value of 21.9 (17.9–23.1) g/serving), followed by protein-rich foods (20.7 (16.8–21.9) g/serving) and fruits and vegetables (16.8 (12.0–17.5) g/serving). Besides significative difference between carbohydrate-rich foods and fruits and vegetables, the two categories exhibited significantly higher median protein content compared to desserts (11.9 (11.0–12.7) g/serving), breakfast meals (7.7 (2.5–10.5) g/serving) and thickened water (0.0 (0.0–0.5) g/serving).

Concerning the fiber content, this was highly variable with a significant amount occurring in carbohydrate-rich foods (total median value of 3.8 (1.5–11.1) g/serving) and protein-rich foods (4.5 (1.6–5.6) g/serving), with no significative differences except for fruits and vegetables (4.0 (3.9–6.0) g/serving). In particular, fruits and vegetables category exhibited a significantly higher fiber content than the desserts (1.9 (1.7–2.6) g/serving), breakfast meals (1.4 (0.8–3.3) g/serving), and thickened water (1.3 (0.9–1.5) g/serving), with the latter being the only category with a median fiber content that is significantly distinct from all other categories.

**Figure 3:**
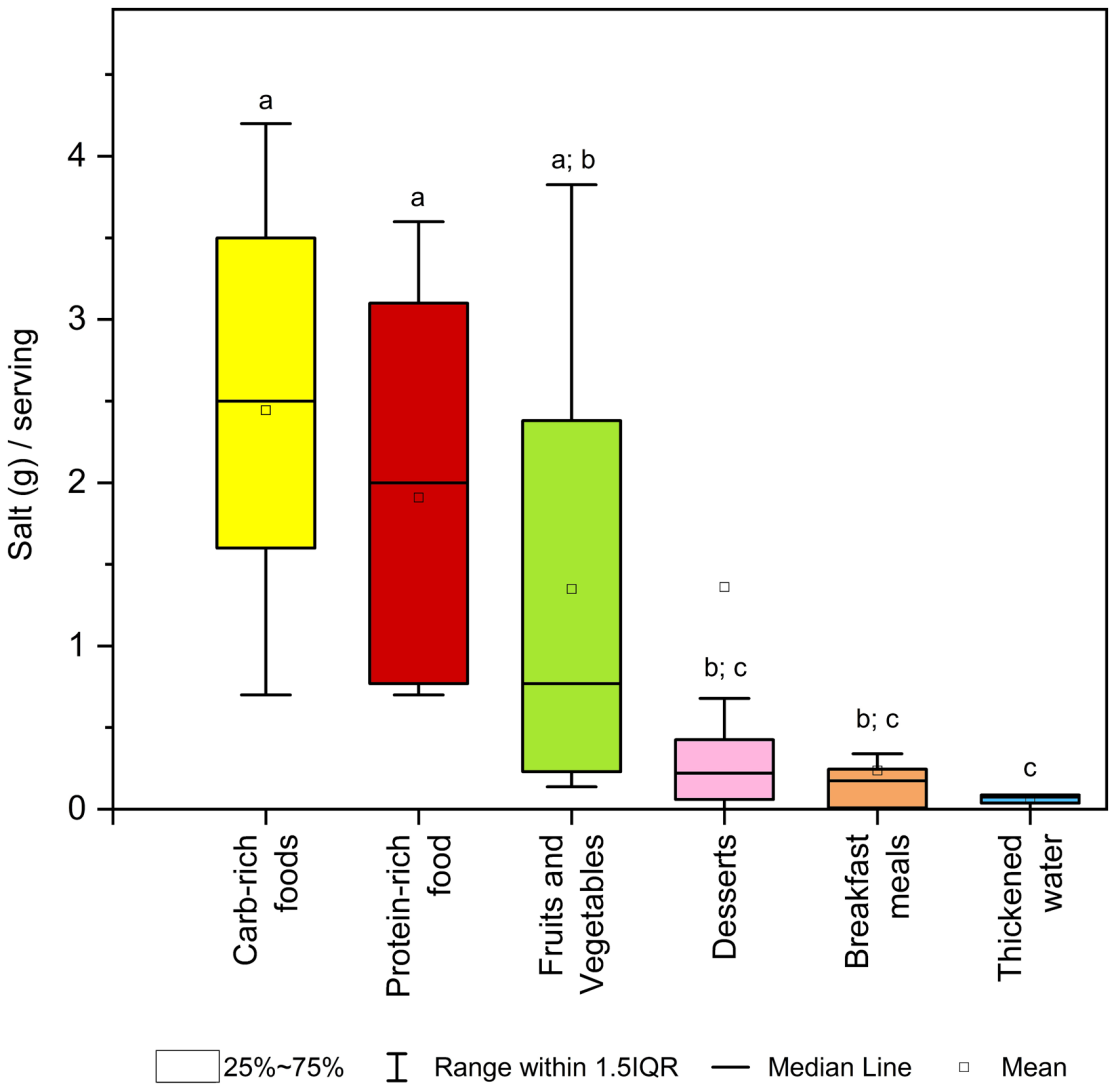
Box plot for salt content (g) per standard serving across categories of dysphagia-oriented products. Standard serving was retrieved for every product as stated on the label. For each category, different upper letters indicate a significant difference among the different dysphagia-oriented products categories (*p* < 0.05; Dunn test). Carb, carbohydrate.

Regarding salt content per serving, the results are shown in Figure 3. Considering the median salt content per standard serving, only desserts (total median value of 0.22 (0.14–0.38) g/serving), breakfast meals (0.18 (0.01–0.25) g/serving) and thickened waters (0.08 (0.06–0.09) g/serving) had significantly lower salt content than the other categories. Carbohydrate-rich foods and protein-rich foods showed high salt per serving, which were (2.5 (1.7–3.4) g/serving) and (2.0 (0.8–3.0) g/serving), respectively, followed by fruits and vegetables (0.77 (0.21–2.38) g/serving).

## 4 Discussion

As far as we know, the present study evaluated, for the first time, the nutritional quality of different dysphagia-oriented products sold on the Italian market, considering the mandatory and some voluntary information reported on the food packages.

One of the most important aspects to be considered in these products is related to the nutritional quality, since people with dysphagia are required to consume a wide range of texture-adapted products which may deeply influence the diet quality and in turn the quality of life. Thus, it is important to know the nutritional characteristics of products on the market, in order to identify potential gaps and criticalities that should be addressed in future formulations.

Nutritional data showed wide differences in energy and nutrients among the six different categories of dysphagia-oriented products under study. Specifically, carbohydrate-rich foods showed the highest values of energy, lipids, SFA, fiber, protein, and salt per 100 g. Conversely, in terms of carbohydrate and sugar content, breakfast substitutes had the highest values per 100 g. Regarding salt content in 100 g, 68% of the products were above the threshold of 0.3 g salt/100 g. Even so, carbohydrate-and protein-rich foods had a very high salt content per 100 g. Specifically, all the two categories had at a minimum median amount of salt twice the identifying value of high-salt products (1 g salt/100 g). These findings are further evident when analyzing the nutrition declaration not per 100 g but per serving, especially since the serving size for all the ready-to-eat products is higher than 100 g. This led to greater differentiation among the different dysphagia-oriented products, with carbohydrate-rich foods showing the highest energy content, although without significant differences with protein-rich foods and fruits and vegetables.

Concerning salt, values per serving confirmed those obtained per 100 g, showing that most of the products currently on the market deeply contribute to salt intake in the dysphagic population. This decision may be related to the fact that consumers are more willing to accept these products when the salt level is appropriate, because salt enhances the flavor of dishes (16). In the present study, a serving of carbohydrates-or protein-rich foods contributes to a high extent to the daily salt intake: on average equivalent to 50% or 40%, respectively, of the 5 g/day indicated as a target by the World Health Organization (WHO) (17). The higher salt content within these categories may be due to the type of meal they replace: main courses are preparations that may require more flavor in order to be more appealing and, as a result, these products may be very enriched with salt. These data suggest the need of reformulating these products to reduce salt intake, the which excessive consumption of salt in the diet may increases blood pressure and consequently the risk of cardiovascular health outcomes (18, 19). Moreover, it is worth to note that, despite the high median salt content retrieved in these products, we also observed a high variability also within the same categories. This suggests that efforts are required to encourage dysphagic people and health professionals to have a proper reading and understanding of food labeling in order to make healthy food choices also in terms of salt intake (20, 21).

Moreover, it should be considered that not only the foods themselves, but also their preparation (e.g., the addition of salt to boiling water), can play a major role in daily nutrient intake. For example, the high salt content in carbohydrate-rich foods that need to be prepared suggests that the addition of salt to the preparation water should be avoided, as well as the use of a broth prepared using salt. In addition, a dysphagic patient’s nutritional team should formulate a diet that avoids sodium-rich foods, as well as a reduction in the amount of salt used in food preparation, thus reducing the value of added sodium in order to maintain or restore the patient’s nutritional status (22). The patient often requires sodium restriction, which should be achieved with a maximum amount of salt equal to 5 g/day (which is equivalent to the value of the entire target). The different levels of restriction are guided by each patient’s pathology and clinical course. Sodium restriction can be mild, moderate, or severe; in the case of moderate restriction, intake should be 2 g/day, while in the case of severe restriction, the patient should not eat added salt, but only the intrinsic salt in his or her food (22). Certainly, this is troublesome if products currently available on the market contain high values of salt. Unfortunately, these turn out to be one of the few foods that are safe for a dysphagic patient to consume, but at the same time they contribute to excessively high amounts of salt for a healthy diet.

As mentioned above, a thorough comparison of current results with previous findings is difficult due to the lack of similar data present in literature. However, some studies have reported some nutritional information such as energy, protein, and lipids (per 100 g) of several commercially-available dysphagia-oriented products. The categories mostly considered were desserts (both mousses and cake substitutes), breakfast meals, and soups. The values for energy, protein, and lipid content per 100 g obtained from our study were in line with values found in the literature (23, 24). While it is noteworthy that there is a lack of specific studies related to salt content within currently marketed products intended for dysphagia.

It is necessary to point out that in almost all labels one of the most important information for the dysphagic patient is absent: the indication of the corresponding level according to the IDDSI scale. This lack is compounded by the complete lack of any information regarding the viscosity of the product, which, in absence of the IDDSI rating, could also help the patient (or clinician) to correctly identify whether that product is suitable for his or her needs or not. In particular, we specify that only four products out of 70 carried the relevant IDDSI level on the label. These products were categorized among fruits and vegetables, and specifically two of these belonged to stage 1 (viscosity range: 1–50 cP), while the others to stage 3 (viscosity range: 351–1750 cP). This significant gap in labeling highlights the crucial need for improved nutritional literacy for dysphagia-oriented products. The absence of serious information such as IDDSI levels and viscosity not only limits the ability of dysphagic patients to make safe and appropriate food choices but also underscores a broader issue of nutritional education. Improving food labels to include at least the IDDSI categorization, alongside comprehensive educational programs, is essential for advancing nutritional literacy. This strategy would ensure that individuals with dysphagia receive the necessary information to make informed dietary choices, ultimately supporting their health and well-being.

There is significant evidence in the scientific literature highlighting the lack of awareness among dysphagic patients regarding the nutritional quality of the foods designed for their condition. Studies indicate that many patients with dysphagia are not adequately informed about the nutritional content of texture-modified foods, which can lead to inadequate dietary intake and increased risk of malnutrition (25). This issue is exacerbated by insufficient labeling and the lack of educational resources provided by food manufacturers and healthcare providers, making it difficult for patients and caregivers to make informed dietary choices. For instance, a comprehensive review on texture-modified foods emphasizes the need for improved nutritional information and guidance to ensure that these foods meet the dietary needs of dysphagic patients (10). Additionally, research has shown that dysphagia can significantly impact nutritional status and increase the risk of malnutrition and depression, further underscoring the importance of nutritional awareness and proper dietary management (26). Addressing these gaps through better labeling practices and targeted educational initiatives could significantly improve the nutritional health and overall well-being of individuals with dysphagia.

This work has shown strengths and limitations, mainly attributable to the methodology used for product selection. Firstly, we analyzed for the first time the nutritional composition of a large number of dysphagia-oriented products retrieved from the main online sales channels of manufacturers or wholesalers dealing with this type of food. On the other hand, the exclusion of products sold by local stores (i.e., pharmacies and stores dedicated to special foods) may have limited the analysis of products. Another limitation of the study relates to the varying origin of the nutrition data reported on the label. These could be based on laboratory analysis or calculations made from the formulation or from generally established and accepted data, creating a potential bias in the origin of the data. In addition, the inability to compare the nutritional quality of dysphagia-oriented products with regular products available on the Italian market represents another limitation, as it could prevent a comprehensive understanding of how these specialized products measure up against standard nutritional benchmarks. Another limitation is related to the fact that for this study only products sold on the Italian market were considered. Indeed, despite the identified products are all produced by leading healthcare and thus can be available also in the EU countries, we cannot exclude that formulation and thus nutritional declaration vary country-by-country taking into consideration also the culturally-different sensory expectations related to the products.

The current market for dysphagia-oriented products is well-established, with multinational healthcare companies investing in their development. However, these products often suffer from poor sensory qualities. Future studies will aim to explore the potential for designing new, and improved products. Given our interest in the upcycling of agri-food waste, we are particularly focused on leveraging this approach to enhance the sensory and nutritional profiles of dysphagia-oriented products. We also aim to explore methods to enhance the sensory qualities of these products while reducing salt content, through innovative food processing techniques.

## 5 Conclusion

To the best of our knowledge, this is the first study to comprehensively analyze the nutritional composition of a wide range of dysphagia-oriented products sold on the Italian market. The data showed that the dysphagia products currently on the market are slightly different in terms of nutritional profile and only partially comparable. In particular, the main courses were characterized by a high salt content per portion, which represents a significant percentage of the maximum daily intake recommended by the WHO. This highlights the need to clarify nutritional data of products to consumers, especially since these are among the few products that can be safely consumed by dysphagic individuals. It is unfortunately contradictory for a patient with swallowing difficulties to ingest over half of the recommended daily salt intake in just one ready meal and then struggle to drink due to their condition. In this regard, it would be advisable to reduce or carefully avoid salt in preparations for dysphagic patients, for example, by not salting the water used to cook dry products or refraining from adding salt to the meal once it is ready. Given the current state of awareness and the nutritional challenges identified, it is evident that comprehensive strategies are needed to address these issues effectively. The convergence of these findings points to a critical need for systemic changes in both labeling practices and educational outreach. Enhanced labels that clearly indicate IDDSI levels, viscosity, and nutritional content, coupled with robust educational initiatives, can empower dysphagic patients and their caregivers. This dual approach would not only promote safer and more informed dietary choices, but also contribute to better health outcomes and quality of life for patients with dysphagia. It is indeed crucial to educate patients on choosing products that meet their physiological needs while providing the right nutrient intake. The goal of dysphagia patient management is not merely to ensure safe food intake, but also to provide optimal nutritional intake, improve the patient’s clinical condition, and ensure that the foods are both satisfying and palatable for the patient.

## Data availability statement

The original contributions presented in the study are included in the article/Supplementary material, further inquiries can be directed to the corresponding author.

## Author contributions

GIA: Formal analysis, Investigation, Methodology, Visualization, Writing – original draft. DM: Conceptualization, Supervision, Writing – review & editing. LP: Conceptualization, Data curation, Funding acquisition, Supervision, Writing – review & editing.
