# Supplementary material for: Nutritional quality of dysphagia-oriented products sold on the Italian market
Source: Front Nutr. 2024 Jul 3;11:1425878. doi: 10.3389/fnut.2024.1425878 (PMC11252055; doi:10.3389/fnut.2024.1425878)
Supplement: Supplementary file 1 [file Table_1.DOCX]

Supplementary Table 1: energy and nutrient content per 100 g of the retrieved products

| **ID code** | **Category** | **Description** | **IDDSI level** [1]  (If reported on the label) | **Energy**  (Kcal/100g) | **Energy**  (KJ/100g) | **Lipids**  (g/100g) | **SFA** (g/100g) | **Carbs** (g/100g) | **Sugars**  (g/100g) | **Fiber**  (g/100g) | **Protein**  (g/100g) | **Salt**  (g/100g) | **Serving size** (g) | **Single portion** |
| --- | --- | --- | --- | --- | --- | --- | --- | --- | --- | --- | --- | --- | --- | --- |
| ID_1 | 1 | Pasta | N.A. | 466 | 1949 | 24 | 18 | 31 | 6,3 | 11 | 25 | 1,6 | 100 | 1 |
| ID_2 | 1 | Pasta | N.A. | 127 | 535 | 5,2 | 1,8 | 13 | 1,1 | 0,5 | 6,9 | 0,25 | 300 | 1 |
| ID_3 | 1 | Rice | N.A. | 436 | 1829 | 16 | 1,8 | 45 | 6,5 | 8 | 24 | 1 | 70 | 0 |
| ID_4 | 1 | Pasta | N.A. | 432 | 1808 | 21 | 3,2 | 33 | 5,2 | 11 | 21 | 3,5 | 100 | 1 |
| ID_5 | 1 | Pasta | N.A. | 390 | 1632 | 15 | 11 | 42 | 14 | 20 | 11 | 2,1 | 100 | 1 |
| ID_6 | 1 | Pasta | N.A. | 415 | 1739 | 17 | 12 | 42 | 22 | 12 | 17 | 2,9 | 100 | 1 |
| ID_7 | 1 | Pasta | N.A. | 458 | 1916 | 16,7 | 7,2 | 48,6 | 3,1 | 1,5 | 27,5 | 3,7 | 100 | 1 |
| ID_8 | 1 | Pasta | N.A. | 460 | 1924 | 16,2 | 3,9 | 55 | 3,5 | 1,1 | 23,1 | 2,5 | 100 | 1 |
| ID_9 | 1 | Rice | N.A. | 458 | 1916 | 16,5 | 3,1 | 53,9 | 5,7 | 1,3 | 22,9 | 2,5 | 100 | 1 |
| ID_10 | 1 | Rice | N.A. | 451 | 1887 | 14,6 | 5,3 | 56,1 | 5,1 | 2 | 22,8 | 4,2 | 100 | 1 |
| ID_11 | 2 | Meat | N.A. | 125 | 525 | 5 | 2,2 | 12,7 | 1,4 | 0,6 | 7 | 0,3 | 300 | 1 |
| ID_12 | 2 | Chicken | N.A. | 132 | 555 | 5,7 | 2,2 | 13,2 | 1,4 | 0,3 | 6,9 | 0,3 | 300 | 1 |
| ID_13 | 2 | Meat | N.A. | 424 | 1781 | 14 | 2 | 47 | 3 | 7 | 24 | 1,1 | 70 | 0 |
| ID_14 | 2 | Legumes | N.A. | 431 | 1808 | 16 | 1,6 | 43 | 3 | 8,5 | 24 | 1,2 | 70 | 0 |
| ID_15 | 2 | Chicken | N.A. | 424 | 1785 | 14 | 1,6 | 46 | 4 | 8,5 | 24 | 1 | 70 | 0 |
| ID_16 | 2 | Meat | N.A. | 427 | 1792 | 15 | 2,4 | 45 | 4 | 8 | 24 | 1 | 70 | 0 |
| ID_17 | 2 | Meat | N.A. | 429 | 1797 | 13 | 1,7 | 51 | 6,5 | 3 | 24 | 1 | 70 | 0 |
| ID_18 | 2 | Chicken | N.A. | 424 | 1785 | 14 | 1,5 | 48 | 5,5 | 8 | 24 | 1,1 | 70 | 0 |
| ID_19 | 2 | Eggs | N.A. | 446 | 1868 | 22 | 14 | 38 | 16 | 6,4 | 19 | 2,3 | 100 | 1 |
| ID_20 | 2 | Eggs | N.A. | 411 | 1718 | 15 | 11 | 43 | 12 | 5,6 | 22 | 2,4 | 100 | 1 |
| ID_21 | 2 | Meat | N.A. | 433 | 1814 | 26 | 19 | 35 | 3,2 | 4,5 | 21 | 1,5 | 100 | 1 |
| ID_22 | 2 | Chicken | N.A. | 469 | 1963 | 27 | 11 | 34 | 12 | 2 | 22 | 2,1 | 100 | 1 |
| ID_23 | 2 | Chicken | N.A. | 443 | 1854 | 26 | 3,6 | 35 | 4,8 | 8,6 | 11 | 3,4 | 100 | 1 |
| ID_24 | 2 | Tuna | N.A. | 458 | 1916 | 15,8 | 7,7 | 51,3 | 5,1 | 5,1 | 25 | 3,1 | 100 | 1 |
| ID_25 | 2 | Chicken | N.A. | 431 | 1803 | 12,4 | 2,6 | 52,3 | 5,8 | 2,8 | 26,1 | 2 | 100 | 1 |
| ID_26 | 2 | Meat | N.A. | 494 | 2067 | 24 | 8,1 | 44,6 | 2,9 | 1,2 | 24,4 | 2,8 | 100 | 1 |
| ID_27 | 2 | Eggs | N.A. | 457 | 1912 | 16,6 | 5,1 | 54,8 | 3,6 | 0,9 | 21,7 | 3,5 | 100 | 1 |
| ID_28 | 2 | Meat | N.A. | 440 | 1841 | 14,5 | 5 | 57,3 | 9,2 | 0,9 | 19,5 | 3,6 | 100 | 1 |
| ID_29 | 2 | Cheese-Eggs | N.A. | 432 | 1808 | 13,2 | 5,1 | 55,9 | 4,7 | 1,4 | 21,7 | 3,3 | 100 | 1 |
| ID_30 | 3 | Vegetables | N.A. | 412 | 1725 | 19 | 17 | 32 | 3,9 | 17 | 18 | 2,8 | 100 | 1 |
| ID_31 | 3 | Vegetables | N.A. | 452 | 1891 | 15,7 | 8,6 | 54 | 5,9 | 6 | 20,7 | 2,6 | 100 | 1 |
| ID_32 | 3 | Vegetables | N.A. | 468 | 1958 | 15,3 | 8,6 | 59,3 | 5,8 | 4,4 | 21 | 0,6 | 85 | 1 |
| ID_33 | 3 | Vegetables | N.A. | 474 | 1983 | 15,4 | 7,4 | 63,4 | 5,2 | 7,4 | 16,7 | 1,4 | 85 | 1 |
| ID_34 | 3 | Vegetables | N.A. | 451 | 1887 | 13,1 | 7,7 | 60,4 | 6,2 | 5,7 | 19,9 | 4,5 | 85 | 1 |
| ID_35 | 3 | Vegetables | N.A. | 454 | 1900 | 15,3 | 6,6 | 57,4 | 5,4 | 4,6 | 19,4 | 2,5 | 85 | 1 |
| ID_36 | 3 | Vegetables | N.A. | 442 | 1848 | 16 | 1,8 | 49 | 2,4 | 1,8 | 25 | 0,86 | 70 | 0 |
| ID_37 | 3 | Vegetables | N.A. | 441 | 1850 | 16 | 1,9 | 46 | 5,5 | 8,5 | 24 | 1,1 | 70 | 0 |
| ID_38 | 3 | Vegetables | N.A. | 433 | 1811 | 13,3 | 3,5 | 56,9 | 4,9 | 1,5 | 20,6 | 2,8 | 85 | 1 |
| ID_39 | 3 | Fruit | 3 | 137 | 580 | 4 | 4 | 17 | 11,3 | 2,6 | 7 | 0,1375 | 150 | 1 |
| ID_40 | 3 | Fruit | 3 | 137 | 575 | 4 | 4 | 17 | 11,3 | 2,6 | 7 | 2,3 | 150 | 1 |
| ID_41 | 3 | Fruit | 1 | 245 | 1030 | 9,3 | 1 | 29,1 | 5,4 | 3,2 | 9,6 | 2,3 | 125 | 1 |
| ID_42 | 3 | Fruit | 1 | 245 | 1030 | 9,3 | 1 | 29,1 | 6,3 | 3,2 | 9,6 | 2,4 | 125 | 1 |
| ID_43 | 4 | Fruit | N.A. | 171 | 716 | 7,35 | 0,85 | 16 | 9 | 1,5 | 9,5 | 0,17 | 125 | 1 |
| ID_44 | 4 | Pudding | N.A. | 170 | 712 | 16 | 9 | 120 | 2 | 15 | 9,5 | 10 | 125 | 1 |
| ID_45 | 4 | Pudding | N.A. | 171 | 716 | 7,35 | 0,85 | 16 | 9 | 1,5 | 9,5 | 0,17 | 125 | 1 |
| ID_46 | 4 | Pudding | N.A. | 71 | 297 | 1,8 | 1,3 | 12 | 9,4 | 2 | 0,9 | 0,06 | 100 | 1 |
| ID_47 | 4 | Pudding | N.A. | 142 | 597 | 4,7 | 1,2 | 14 | 3 | 2 | 8,8 | 2,1 | 125 | 1 |
| ID_48 | 4 | Pudding | N.A. | 142 | 597 | 4,7 | 1,2 | 14 | 3 | 2 | 8,8 | 2,1 | 125 | 1 |
| ID_49 | 4 | Pudding | N.A. | 360 | 1508 | 0,8 | 0,2 | 41 | 16 | 6,6 | 44 | 1,3 | 25 | 1 |
| ID_50 | 4 | Cake | N.A. | 464 | 1941 | 17,7 | 5,8 | 57,9 | 7,8 | 3,1 | 18,3 | 0,8 | 85 | 1 |
| ID_51 | 4 | Cake | N.A. | 477 | 1997 | 18,9 | 2,6 | 56,3 | 10,5 | 3,2 | 20,5 | 0,5 | 85 | 1 |
| ID_52 | 4 | Cake | N.A. | 479 | 2004 | 19,4 | 9,3 | 60,1 | 11,3 | 3,1 | 16 | 0,4 | 85 | 1 |
| ID_53 | 4 | Cake | N.A. | 479 | 2004 | 19,4 | 9,3 | 60,1 | 11,3 | 3,1 | 16 | 0,4 | 55 | 1 |
| ID_54 | 5 | Milk-Cereal | N.A. | 347 | 1465 | 2,2 | 0,4 | 67 | 7,2 | 11 | 9 | 0,029 | 30 | 0 |
| ID_55 | 5 | Milk-Cereal | N.A. | 375 | 1592 | 1 | 0,2 | 83 | 13 | 2 | 8 | 0,04 | 30 | 0 |
| ID_56 | 5 | Milk-Cereal | N.A. | 377 | 1602 | 1,2 | 0,25 | 83 | 11 | 1,3 | 8,4 | 0,03 | 30 | 0 |
| ID_57 | 5 | Milk-Cereal | N.A. | 371 | 1575 | 1,1 | 0,22 | 82 | 22 | 2,6 | 7,2 | 0,025 | 30 | 0 |
| ID_58 | 5 | Milk-Cereal | N.A. | 432 | 1818 | 11,5 | 1,5 | 58 | 3 | 4 | 22 | 0,5 | 35 | 0 |
| ID_59 | 5 | Milk-Cereal | N.A. | 420 | 1757 | 10 | 2,2 | 65 | 17 | 5 | 15 | 0,34 | 70 | 0 |
| ID_60 | 5 | Milk-Cereal | N.A. | 416 | 1741 | 9 | 4,5 | 68 | 33 | 2,5 | 15 | 0,35 | 70 | 0 |
| ID_61 | 5 | Milk-Cereal | N.A. | 441 | 1845 | 16,8 | 3,2 | 53,3 | 9,2 | 5,8 | 19,1 | 0,4 | 85 | 1 |
| ID_62 | 5 | Milk-Cereal | N.A. | 392 | 1646 | 8 | 4,7 | 60 | 39 | 0,9 | 19 | 1,1 | 100 | 1 |
| ID_63 | 6 | Water | N.A. | 6 | 25 | 0 | 0 | 1,2 | 0 | 0,6 | 0 | 0,06 | 125 | 1 |
| ID_64 | 6 | Water | N.A. | 6 | 25 | 0 | 0 | 1 | 0 | 0,7 | 0 | 0,06 | 125 | 1 |
| ID_65 | 6 | Water | N.A. | 6 | 25 | 0 | 0 | 1 | 0 | 0,7 | 0 | 0,06 | 125 | 1 |
| ID_66 | 6 | Water | N.A. | 4 | 15 | 0,5 | 0,1 | 0,5 | 0,5 | 1,2 | 0,5 | 0,07 | 125 | 1 |
| ID_67 | 6 | Water | N.A. | 0,8 | 3 | 0 | 0 | 0,2 | 0 | 1 | 0 | 0,05 | 125 | 1 |
| ID_68 | 6 | Water | N.A. | 29 | 122 | 0 | 0 | 7,2 | 0 | 1 | 0 | 0,05 | 125 | 1 |
| ID_69 | 6 | Water | N.A. | 3,7 | 15,2 | 0,5 | 0 | 0,5 | 0,5 | 1,2 | 0,5 | 0,07 | 125 | 0 |
| ID_70 | 6 | Water | N.A. | 3,7 | 15,2 | 0,5 | 0 | 0,5 | 0,5 | 1,2 | 0,5 | 0,07 | 125 | 0 |

Serving size is given for each product and whether the product is single-portioned (1) or not (0).

The categories are as follows: 1) carbohydrates-rich foods; 2) protein-rich foods; 3) fruits and vegetables; 4) desserts; 5) breakfast meals; 6) thickened water.

N.A. = data not available (not reported on the label and/or on manufacture’s website).

Supplementary Table 2: energy and nutrient content per serving of the retrieved products

| **ID code** | **Category** | **Description** | **IDDSI level** [1]  (If reported on the label) | **Energy**  (Kcal/100g) | **Energy**  (KJ/100g) | **Lipids**  (g/100g) | **SFA** (g/100g) | **Carbs** (g/100g) | **Sugars**  (g/100g) | **Fiber**  (g/100g) | **Protein**  (g/100g) | **Salt**  (g/100g) | **Serving size** (g) | **Single portion** |
| --- | --- | --- | --- | --- | --- | --- | --- | --- | --- | --- | --- | --- | --- | --- |
| ID_1 | 1 | Pasta | N.A. | 466 | 1949 | 24 | 18 | 31 | 6,3 | 11 | 25 | 1,6 | 100 | 1 |
| ID_2 | 1 | Pasta | N.A. | 127 | 535 | 5,2 | 1,8 | 13 | 1,1 | 0,5 | 6,9 | 0,25 | 300 | 1 |
| ID_3 | 1 | Rice | N.A. | 436 | 1829 | 16 | 1,8 | 45 | 6,5 | 8 | 24 | 1 | 70 | 0 |
| ID_4 | 1 | Pasta | N.A. | 432 | 1808 | 21 | 3,2 | 33 | 5,2 | 11 | 21 | 3,5 | 100 | 1 |
| ID_5 | 1 | Pasta | N.A. | 390 | 1632 | 15 | 11 | 42 | 14 | 20 | 11 | 2,1 | 100 | 1 |
| ID_6 | 1 | Pasta | N.A. | 415 | 1739 | 17 | 12 | 42 | 22 | 12 | 17 | 2,9 | 100 | 1 |
| ID_7 | 1 | Pasta | N.A. | 458 | 1916 | 16,7 | 7,2 | 48,6 | 3,1 | 1,5 | 27,5 | 3,7 | 100 | 1 |
| ID_8 | 1 | Pasta | N.A. | 460 | 1924 | 16,2 | 3,9 | 55 | 3,5 | 1,1 | 23,1 | 2,5 | 100 | 1 |
| ID_9 | 1 | Rice | N.A. | 458 | 1916 | 16,5 | 3,1 | 53,9 | 5,7 | 1,3 | 22,9 | 2,5 | 100 | 1 |
| ID_10 | 1 | Rice | N.A. | 451 | 1887 | 14,6 | 5,3 | 56,1 | 5,1 | 2 | 22,8 | 4,2 | 100 | 1 |
| ID_11 | 2 | Meat | N.A. | 125 | 525 | 5 | 2,2 | 12,7 | 1,4 | 0,6 | 7 | 0,3 | 300 | 1 |
| ID_12 | 2 | Chicken | N.A. | 132 | 555 | 5,7 | 2,2 | 13,2 | 1,4 | 0,3 | 6,9 | 0,3 | 300 | 1 |
| ID_13 | 2 | Meat | N.A. | 424 | 1781 | 14 | 2 | 47 | 3 | 7 | 24 | 1,1 | 70 | 0 |
| ID_14 | 2 | Legumes | N.A. | 431 | 1808 | 16 | 1,6 | 43 | 3 | 8,5 | 24 | 1,2 | 70 | 0 |
| ID_15 | 2 | Chicken | N.A. | 424 | 1785 | 14 | 1,6 | 46 | 4 | 8,5 | 24 | 1 | 70 | 0 |
| ID_16 | 2 | Meat | N.A. | 427 | 1792 | 15 | 2,4 | 45 | 4 | 8 | 24 | 1 | 70 | 0 |
| ID_17 | 2 | Meat | N.A. | 429 | 1797 | 13 | 1,7 | 51 | 6,5 | 3 | 24 | 1 | 70 | 0 |
| ID_18 | 2 | Chicken | N.A. | 424 | 1785 | 14 | 1,5 | 48 | 5,5 | 8 | 24 | 1,1 | 70 | 0 |
| ID_19 | 2 | Eggs | N.A. | 446 | 1868 | 22 | 14 | 38 | 16 | 6,4 | 19 | 2,3 | 100 | 1 |
| ID_20 | 2 | Eggs | N.A. | 411 | 1718 | 15 | 11 | 43 | 12 | 5,6 | 22 | 2,4 | 100 | 1 |
| ID_21 | 2 | Meat | N.A. | 433 | 1814 | 26 | 19 | 35 | 3,2 | 4,5 | 21 | 1,5 | 100 | 1 |
| ID_22 | 2 | Chicken | N.A. | 469 | 1963 | 27 | 11 | 34 | 12 | 2 | 22 | 2,1 | 100 | 1 |
| ID_23 | 2 | Chicken | N.A. | 443 | 1854 | 26 | 3,6 | 35 | 4,8 | 8,6 | 11 | 3,4 | 100 | 1 |
| ID_24 | 2 | Tuna | N.A. | 458 | 1916 | 15,8 | 7,7 | 51,3 | 5,1 | 5,1 | 25 | 3,1 | 100 | 1 |
| ID_25 | 2 | Chicken | N.A. | 431 | 1803 | 12,4 | 2,6 | 52,3 | 5,8 | 2,8 | 26,1 | 2 | 100 | 1 |
| ID_26 | 2 | Meat | N.A. | 494 | 2067 | 24 | 8,1 | 44,6 | 2,9 | 1,2 | 24,4 | 2,8 | 100 | 1 |
| ID_27 | 2 | Eggs | N.A. | 457 | 1912 | 16,6 | 5,1 | 54,8 | 3,6 | 0,9 | 21,7 | 3,5 | 100 | 1 |
| ID_28 | 2 | Meat | N.A. | 440 | 1841 | 14,5 | 5 | 57,3 | 9,2 | 0,9 | 19,5 | 3,6 | 100 | 1 |
| ID_29 | 2 | Cheese-Eggs | N.A. | 432 | 1808 | 13,2 | 5,1 | 55,9 | 4,7 | 1,4 | 21,7 | 3,3 | 100 | 1 |
| ID_30 | 3 | Vegetables | N.A. | 412 | 1725 | 19 | 17 | 32 | 3,9 | 17 | 18 | 2,8 | 100 | 1 |
| ID_31 | 3 | Vegetables | N.A. | 452 | 1891 | 15,7 | 8,6 | 54 | 5,9 | 6 | 20,7 | 2,6 | 100 | 1 |
| ID_32 | 3 | Vegetables | N.A. | 468 | 1958 | 15,3 | 8,6 | 59,3 | 5,8 | 4,4 | 21 | 0,6 | 85 | 1 |
| ID_33 | 3 | Vegetables | N.A. | 474 | 1983 | 15,4 | 7,4 | 63,4 | 5,2 | 7,4 | 16,7 | 1,4 | 85 | 1 |
| ID_34 | 3 | Vegetables | N.A. | 451 | 1887 | 13,1 | 7,7 | 60,4 | 6,2 | 5,7 | 19,9 | 4,5 | 85 | 1 |
| ID_35 | 3 | Vegetables | N.A. | 454 | 1900 | 15,3 | 6,6 | 57,4 | 5,4 | 4,6 | 19,4 | 2,5 | 85 | 1 |
| ID_36 | 3 | Vegetables | N.A. | 442 | 1848 | 16 | 1,8 | 49 | 2,4 | 1,8 | 25 | 0,86 | 70 | 0 |
| ID_37 | 3 | Vegetables | N.A. | 441 | 1850 | 16 | 1,9 | 46 | 5,5 | 8,5 | 24 | 1,1 | 70 | 0 |
| ID_38 | 3 | Vegetables | N.A. | 433 | 1811 | 13,3 | 3,5 | 56,9 | 4,9 | 1,5 | 20,6 | 2,8 | 85 | 1 |
| ID_39 | 3 | Fruit | 3 | 137 | 580 | 4 | 4 | 17 | 11,3 | 2,6 | 7 | 0,1375 | 150 | 1 |
| ID_40 | 3 | Fruit | 3 | 137 | 575 | 4 | 4 | 17 | 11,3 | 2,6 | 7 | 2,3 | 150 | 1 |
| ID_41 | 3 | Fruit | 1 | 245 | 1030 | 9,3 | 1 | 29,1 | 5,4 | 3,2 | 9,6 | 2,3 | 125 | 1 |
| ID_42 | 3 | Fruit | 1 | 245 | 1030 | 9,3 | 1 | 29,1 | 6,3 | 3,2 | 9,6 | 2,4 | 125 | 1 |
| ID_43 | 4 | Fruit | N.A. | 171 | 716 | 7,35 | 0,85 | 16 | 9 | 1,5 | 9,5 | 0,17 | 125 | 1 |
| ID_44 | 4 | Pudding | N.A. | 170 | 712 | 16 | 9 | 120 | 2 | 15 | 9,5 | 10 | 125 | 1 |
| ID_45 | 4 | Pudding | N.A. | 171 | 716 | 7,35 | 0,85 | 16 | 9 | 1,5 | 9,5 | 0,17 | 125 | 1 |
| ID_46 | 4 | Pudding | N.A. | 71 | 297 | 1,8 | 1,3 | 12 | 9,4 | 2 | 0,9 | 0,06 | 100 | 1 |
| ID_47 | 4 | Pudding | N.A. | 142 | 597 | 4,7 | 1,2 | 14 | 3 | 2 | 8,8 | 2,1 | 125 | 1 |
| ID_48 | 4 | Pudding | N.A. | 142 | 597 | 4,7 | 1,2 | 14 | 3 | 2 | 8,8 | 2,1 | 125 | 1 |
| ID_49 | 4 | Pudding | N.A. | 360 | 1508 | 0,8 | 0,2 | 41 | 16 | 6,6 | 44 | 1,3 | 25 | 1 |
| ID_50 | 4 | Cake | N.A. | 464 | 1941 | 17,7 | 5,8 | 57,9 | 7,8 | 3,1 | 18,3 | 0,8 | 85 | 1 |
| ID_51 | 4 | Cake | N.A. | 477 | 1997 | 18,9 | 2,6 | 56,3 | 10,5 | 3,2 | 20,5 | 0,5 | 85 | 1 |
| ID_52 | 4 | Cake | N.A. | 479 | 2004 | 19,4 | 9,3 | 60,1 | 11,3 | 3,1 | 16 | 0,4 | 85 | 1 |
| ID_53 | 4 | Cake | N.A. | 479 | 2004 | 19,4 | 9,3 | 60,1 | 11,3 | 3,1 | 16 | 0,4 | 55 | 1 |
| ID_54 | 5 | Milk-Cereal | N.A. | 347 | 1465 | 2,2 | 0,4 | 67 | 7,2 | 11 | 9 | 0,029 | 30 | 0 |
| ID_55 | 5 | Milk-Cereal | N.A. | 375 | 1592 | 1 | 0,2 | 83 | 13 | 2 | 8 | 0,04 | 30 | 0 |
| ID_56 | 5 | Milk-Cereal | N.A. | 377 | 1602 | 1,2 | 0,25 | 83 | 11 | 1,3 | 8,4 | 0,03 | 30 | 0 |
| ID_57 | 5 | Milk-Cereal | N.A. | 371 | 1575 | 1,1 | 0,22 | 82 | 22 | 2,6 | 7,2 | 0,025 | 30 | 0 |
| ID_58 | 5 | Milk-Cereal | N.A. | 432 | 1818 | 11,5 | 1,5 | 58 | 3 | 4 | 22 | 0,5 | 35 | 0 |
| ID_59 | 5 | Milk-Cereal | N.A. | 420 | 1757 | 10 | 2,2 | 65 | 17 | 5 | 15 | 0,34 | 70 | 0 |
| ID_60 | 5 | Milk-Cereal | N.A. | 416 | 1741 | 9 | 4,5 | 68 | 33 | 2,5 | 15 | 0,35 | 70 | 0 |
| ID_61 | 5 | Milk-Cereal | N.A. | 441 | 1845 | 16,8 | 3,2 | 53,3 | 9,2 | 5,8 | 19,1 | 0,4 | 85 | 1 |
| ID_62 | 5 | Milk-Cereal | N.A. | 392 | 1646 | 8 | 4,7 | 60 | 39 | 0,9 | 19 | 1,1 | 100 | 1 |
| ID_63 | 6 | Water | N.A. | 6 | 25 | 0 | 0 | 1,2 | 0 | 0,6 | 0 | 0,06 | 125 | 1 |
| ID_64 | 6 | Water | N.A. | 6 | 25 | 0 | 0 | 1 | 0 | 0,7 | 0 | 0,06 | 125 | 1 |
| ID_65 | 6 | Water | N.A. | 6 | 25 | 0 | 0 | 1 | 0 | 0,7 | 0 | 0,06 | 125 | 1 |
| ID_66 | 6 | Water | N.A. | 4 | 15 | 0,5 | 0,1 | 0,5 | 0,5 | 1,2 | 0,5 | 0,07 | 125 | 1 |
| ID_67 | 6 | Water | N.A. | 0,8 | 3 | 0 | 0 | 0,2 | 0 | 1 | 0 | 0,05 | 125 | 1 |
| ID_68 | 6 | Water | N.A. | 29 | 122 | 0 | 0 | 7,2 | 0 | 1 | 0 | 0,05 | 125 | 1 |
| ID_69 | 6 | Water | N.A. | 3,7 | 15,2 | 0,5 | 0 | 0,5 | 0,5 | 1,2 | 0,5 | 0,07 | 125 | 0 |
| ID_70 | 6 | Water | N.A. | 3,7 | 15,2 | 0,5 | 0 | 0,5 | 0,5 | 1,2 | 0,5 | 0,07 | 125 | 0 |

The categories are as follows: 1) carbohydrates-rich foods; 2) protein-rich foods; 3) fruits and vegetables; 4) desserts; 5) breakfast meals; 6) thickened water.

N.A. = data not available (not reported on the label and/or on manufacture’s website).
